# Precision Workforce Management for Radiographers: Monitoring and Managing Competences with an Automatic Tool

**DOI:** 10.3390/jpm14070669

**Published:** 2024-06-21

**Authors:** Andrea Lastrucci, Yannick Wandael, Giovanni Orlandi, Angelo Barra, Stefano Chiti, Valentina Gigli, Massimo Marletta, Davide Pelliccia, Barbara Tonietti, Renzo Ricci, Daniele Giansanti

**Affiliations:** 1 Department of Allied Health Professions, Azienda Ospedaliero-Universitaria Careggi, 50134 Florence, Italy; andrea.lastrucci@unifi.it (A.L.); wandaely@aou-careggi.toscana.it (Y.W.); orlandigi@aou-careggi.toscana.it (G.O.); barraa@aou-careggi.toscana.it (A.B.); chitis@aou-careggi.toscana.it (S.C.); riccire@aou-careggi.toscana.it (R.R.); 2 Staff della Direzione Aziendale, Azienda Ospedaliero-Universitaria Careggi, 50134 Florence, Italy; gigliv@aou-careggi.toscana.it (V.G.); toniettib@aou-careggi.toscana.it (B.T.); 3 Department of Allied Health Professions, Azienda Ospedaliero-Universitaria Pisana, 56124 Pisa, Italy; m.marletta@ao-pisa.toscana.it (M.M.); d.pelliccia@ao-pisa.toscana.it (D.P.); 4 Centre TISP, Istituto Superiore di Sanità, 00161 Roma, Italy

**Keywords:** radiographer, skill, competence, workload, performance, monitoring

## Abstract

Optimizing work shifts in healthcare is crucial for maintaining high standards of service delivery and fostering professional development. This study delves into the emerging field of skill-oriented work shift optimization, focusing specifically on radiographers within the healthcare sector. Through the development of Skills Retention Monitoring (SRH), this research aims to enhance skill monitoring, workload management, and organizational performance. In this study, several key highlights emerged: (a) Introduction of the SRH tool: The SRH tool represents a resource-efficient solution that harnesses existing software infrastructure. A preliminary version, focusing on the radiographers’ professional profile, was released, and after several months of use, it demonstrated effectiveness in optimizing work based on competency monitoring. (b) The SRH tool has thus demonstrated the capacity to generate actionable insights in the organizational context of radiographers. By generating weekly reports, the SRH tool streamlines activity management and optimizes resource allocation within healthcare settings. (c) Application of a Computer-Assisted Web Interviewing (CAWI) tool for pre-release feedback during a training event. (d) Strategic importance of a maintenance and monitoring plan: This plan, rooted in a continuous quality improvement approach and key performance indicators, ensures the sustained effectiveness of the SRH tool. (e) Strategic importance of a transfer plan: Involving professional associations and employing targeted questionnaires, this plan ensures the customization of the tool from the perspective of each profession involved. This is a crucial point, as it will enable the release of tool versions tailored to various professions operating within the hospital sector. As a side result, the tool could allow for a more tailored and personalized medicine both by connecting the insights gathered through the SRH tool with the right competencies for healthcare professionals and with individual patient data. This integration could lead to better-informed decision making, optimizing treatment strategies based on both patient needs and the specific expertise of the healthcare provider. Future directions include deploying the SRH tool within the Pisa hospital network and exploring integration with AI algorithms for further optimization. Overall, this research contributes to advancing work shift optimization strategies and promoting excellence in healthcare service delivery.

## 1. Introduction

### 1.1. Background

The competence and ability of radiographers are crucial to patient care [1], extending far beyond mere technical knowledge to encompass a holistic approach that includes patient-centered care and collaboration with other healthcare professionals [2,3]. In particular, the study by Hyde and Hardy [4] highlights the need for further work to identify measurable service delivery outcomes that represent patient-centered care within radiographic practice. In the literature, there is ample evidence emphasizing the significance of assessing radiographers’ clinical competence as a fundamental aspect of upholding professional standards across various radiography activities. This includes Magnetic Resonance Imaging (MRI) examinations [5], the evaluation of image-guided radiotherapy [6], and the reporting of plain radiographs in clinical practice [7]. Enhancing radiographers’ competencies, particularly in advanced imaging technologies, necessitates a consensus on education, training, and competencies within the workforce. This reflects an organizational commitment to continuous development and technological advancements [8]. So, the organizational strategies that promote a supportive work environment, interprofessional collaboration, and access to educational resources are crucial. Regarding this, the importance of an effective work shift organization has been increasingly recognized in healthcare settings for its profound impact on staff well-being and patient safety outcomes and care quality [9,10,11]. Numerous methodologies and techniques have been employed and described in the literature to enhance work organization in healthcare. These strategies focus on improving efficiency, the quality of patient care, and staff satisfaction by making management decisions such as the use of technology to manage open shifts in nursing [12] and the use of variable shift systems through flexible scheduling and strategic break times for nurses working extended day shifts [13]. These findings highlight the importance for healthcare organizations to implement innovative and efficient strategies to ensure a healthy and productive work environment maintaining healthcare competencies. The development of competency frameworks in healthcare is vital for defining a competent workforce, facilitating mobility, and assessing expertise. A recent scoping review found a significant diversity in the methods used to develop these frameworks, with no standard approach identified [14]. This variability indicates a need for improved guidance in developing and reporting competency frameworks to ensure they are fit for purpose. In recent years, there has been a growing interest in optimizing and/or investigating work shifts in healthcare settings based advanced technologies like the development of customized software [15,16] and also wearable technologies [16].

The optimization of work shifts in healthcare thus involves a multifaceted approach that incorporates technological advancements and workers’ competencies and their physiological and psychological well-being. By adopting such an approach, healthcare facilities can enhance both technical skills and patient outcomes.

References [15,16] were not primarily designed to monitor and develop competencies based on work shifts. While they offer valuable insights into related areas, such as staff scheduling and physical activity levels, they do not directly address the systematic tracking or enhancement of skills and competencies associated with shift work.

These references underscore the significance of developing tailored software solutions to meet the unique demands of various workplaces. The first study, [15], delves into the development of a personalized staff scheduling method aimed at improving the work–life balance of hospital employees. Although its focus lies on shift scheduling, it highlights the critical need for customized software solutions to effectively address the complex organizational requirements of shift-based industries. It is important to note, however, that this study does not delve into the specifics of competency monitoring or development.

Similarly, the second study, [16], examines the physical activity levels of hospital shift workers, providing valuable insights into the health implications of shift work. While it does not directly explore software development, it indirectly underscores the importance of tailored interventions to cater to the unique needs of shift workers.

While both studies emphasize the necessity of customized software solutions to tackle specific workplace challenges and enhance employee well-being, they do not directly delve into the realm of competency monitoring or development. This underscores the potential for further research and innovation in this area to optimize the effectiveness of shift work arrangements.

In the development of specialized software tailored for monitoring and enhancing competencies within hospital shift work processes, it is imperative to prioritize both creative design and personalized features that align closely with the unique requirements of the healthcare environment. This involves crafting solutions that not only address the specific challenges of managing shifts and optimizing workflow but also seamlessly integrate with existing organizational systems and practices. By ensuring a high degree of customization and adaptability, such software can effectively support the diverse needs of healthcare professionals and contribute to improved efficiency and performance within hospital settings. Moreover, given the prevailing emphasis on cost effectiveness in today’s operational landscape, there is a pressing need to adopt strategies for minimizing expenses associated with software development. This entails exploring avenues for low-cost development approaches while still maintaining the quality and functionality of the software. By adopting lean development methodologies, leveraging open-source technologies, and prioritizing essential features, organizations can mitigate the financial burden often associated with software projects. This not only facilitates broader accessibility to these tools but also allows for more sustainable long-term utilization, ultimately enhancing the overall value proposition of competency monitoring and development solutions in hospital settings.

### 1.2. Introduction to the Study’s Relevance

The clinical experience is essential for achieving the highest level of clinical competence [17]. Specific requirements, such as the number of examinations performed to achieve certain levels of clinical competence, are outlined in various studies [18].

To gain a comprehensive understanding of the importance of “clinical competence” within the global context, we explored the broader landscape of international research. Using PubMed, we employed a set of composite keywords, as detailed in Box S1 in the Supplementary Materials. Our investigation revealed significant trends in the workload, competencies, and performance of healthcare professionals, depicted in Figures S1–S3 (Supplementary Materials). These figures highlight a notable surge of interest over the past five years, particularly in light of the global pandemic.

Figure S1 illustrates a steady rise in attention to workload, capturing 64% of total publications, with the first study published in 2008 [19]. Similarly, Figure S2 shows a parallel increase in interest regarding healthcare professionals’ competencies, a subject accounting for 59% of publications in recent years, with initial studies dating back to 1991 [20]. Figure S3 highlights a significant focus on performance, emphasizing effectiveness and efficiency and constituting 56% of publications in the last five years [21].

This burgeoning interest underscores two vital aspects: first, the indispensable role of healthcare professionals in navigating the complexities of the pandemic and second, the growing attention towards optimizing their work dynamics. The trends depicted in the data emphasize the critical need to understand and enhance the workload, competencies, and performance of healthcare professionals.

The strategic importance of competence in managing workload and performance is crucial for ensuring patient safety during professional practice [10,11]. Competent healthcare professionals are better equipped to handle the demands of their workload efficiently and safely, thereby enhancing overall performance and ensuring the highest standards of care [9]. Given this critical interconnection, there is an urgent need for comprehensive studies to examine professional skills and competencies and their impact on workload, performance, and safety in healthcare settings. Such studies are necessary to develop targeted interventions that can optimize these factors, ultimately leading to improved healthcare outcomes and safer patient care.

### 1.3. Aim

The department of Allied Health Professions of Careggi Hospital includes 12 different healthcare professional profiles. One of the principal commitments of the Allied Health Professions Department at Careggi Hospital is centered on maintaining a high level of technical competence in the execution of MRI and Computed Tomography (CT). In line with this commitment, the main aim of this study was to design and develop an automatic tool named “Skill Retention Healthcare” (SRH), specifically tailored to optimize daily work shift planning for CT and MRI procedures. This tool was envisioned to play a critical role in maintaining and enhancing the technical competence of healthcare professionals (radiographers) involved in MRI and CT exams within the Allied Health Professions Department at Careggi Hospital.

#### Subsidiary Goals

The first dissemination of the SRH tool in a training event and structured feedback using a CAWI within the department before the release.

Definition of the release of the maintenance, monitoring, and transfer plan of the SRH tool: the maintenance, monitoring, and transfer plan of the SRH tool is strategic because it ensures continuous functionality and reliability while facilitating the tool’s adaptation across different healthcare professions, thereby enhancing overall healthcare service quality and efficiency.

An indirect subsidiary goal is to provide a tool that could allow for more tailored and personalized medicine both by connecting the insights gathered through the SRH and CAWI tools with the right competencies for healthcare professionals and with individual patient data.

## 2. Methods

A literature review was conducted to define the necessary methodology for determining the required competence levels for radiographers. It was found that clinical experience is essential for achieving the highest level of clinical competence [17] and that specific requirements, such as the number of examinations performed to achieve certain levels of clinical competence, are described. The core of the SRH tool is the number of CT and MRI examinations that each radiographer performs within a specified timeframe. To determine the number of examinations required to achieve competency and autonomy, a focus group was formed. Within this focus group, the MRI and CT specialists worked with the coordinator to define the “target ranges”, which represent the optimal number of examinations performed for each macro-area and include multiple CT or MRI procedures required for competency. This information is contained in the Radiological Information System (RIS), which contains data on the number and type of CT or MRI examinations performed by a radiographer within a given period. Indeed, each radiographer is required to record the performed examinations within the RIS. Each radiographer belongs to a specific “functional area” within the department that determines which coordinator oversees their activities. These details are documented in the specialized personnel management software used at Careggi Hospital. The development of the SRH tool is based on the data retrieved and collated from these two software systems for each radiographer. In this study, SAP Business Objects (v.12.1.0.) software is used to retrieve the data from the RIS and the personnel management software. SAP Business Objects is known for its robust business intelligence capabilities and enables in-depth data analysis, ensuring that comprehensive insights can be gained from the combined data sets.

The utilization of the SRH tool was introduced in a departmental course attended in March 2024 by all coordinators associated with Department of Allied Health Professions, aiming to evaluate its acceptance and applicability in various professional environments. Following the presentation, a focus group was activated with a discussion. An invitation to complete an anonymous electronic questionnaire was sent to all coordinators who participated in the departmental course and the focus group. The electronic questionnaire effectively served as a virtual instrument for gathering structured opinions from the focus group during the training event before the release. In particular, an anonymous survey was conducted using a state-of-the-art CAWI tool. Microsoft Forms was used in the development of this CAWI tool, which was specifically chosen for its seamless integration with Office 365 (version 2024). These strategic choices regarding participants and data collection tools were carefully made to ensure the efficiency, reliability, and ethical integrity of the research process. The CAWI instrument was used in a peer-to-peer process to ensure anonymity of respondents. 

The survey after the training event consisted of 18 questions, including 15 single-choice questions and 3 questions that used a Likert scale with a 5-point rating. The key elements requested in the survey are summarized in Table 1.

In developing the release, maintenance, and monitoring plan, we employed the concept of continuous quality improvement applied within the national healthcare system. This approach includes monitoring actions, targeted feedback, and counteractions based on specialized surveys tailored to the various stakeholders involved. Additionally, to obtain objective indicators regarding the added value, effectiveness of the tool, impact on safety, communication chain, and impact on the development of the internal leadership and team empowerment, we proposed an integrative research contribution complementary to the quality control approach. This innovative contribution was based on the selection of appropriate key performance indicators derived from the scientific literature. In defining the transfer plan, we considered the necessity of initiating targeted interactions with professional associations and the involved professionals, as well as the need to develop suitable tools to gather useful feedback for the specific and targeted upgrading of the SRH tool for each profession. The methodology included the definition of a roadmap where questionnaires played a central role. 

## 3. Results 

The results are organized into sections and subsections.

Section 3.1 “Utilization of a SRH Tool for Optimizing Daily Work Shift Planning in CT and MRI: From Development to Clinical Implementation” provides an overview of the tool’s evolution from its inception to its integration into clinical practice.

Section 3.2 “Findings from the training event” outlines the results obtained from administering the electronic survey to multi-professional coordinators within the Department of Allied Health Professions at Careggi Hospital during the training event before the SRH tool release. 

Section 3.3 “Comprehensive SRH Tool Release, Maintenance, Monitoring and Transfer Plan” outlines a detailed strategy for the release, upkeep, surveillance, and relocation of the SRH Tool.

### 3.1. Utilization of a SRH Tool for Optimizing Daily Work Shift Planning in CT and MRI: From Development to Clinical Implementation

**Figure 1:**
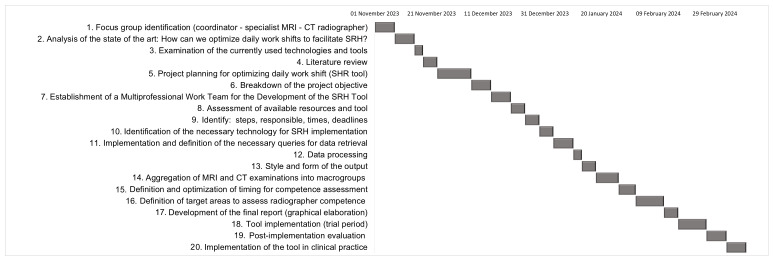
Gantt chart illustrating all phases from creation to implementation of the SRH tool.

The implementation of the SRH tool in clinical practice required over 4 months of work (130 working days in total). Figure 1 shows a Gantt chart with the tasks and their durations from the creation to the use of the SRH tool in clinical practice. In particular, it shows the durations of the 19 identified tasks. The task that took the most time was “Project planning to optimize daily work shifts” (14 days). This activity involved a detailed study of the development of the project and included brainstorming sessions between the focus group members and the staff of the Department of Allied Health Professions at Careggi Hospital. 

Two additional phases that had a short duration but played a key role in the evaluation of data obtained from the SRH tool were the tasks related to:

- Aggregation of CT and MRI examinations into macro-groups (8 days). In this case, the task of the focus group was to identify the different and numerous MRI and CT examinations performed and recorded by the radiographers in the RIS and to summarize them in macro groups defined according to the anatomical district. In particular, 108 different CT examinations were categorized into five macro-groups and 137 MRI examinations into nine macro-groups.
- Definition of target ranges to assess radiographer competence (10 days). In this activity, the focus group defined the “target ranges”, which represent the number of examinations (±15%) for each macro group that each radiographer must perform within a given timeframe to maintain their competence. If the number of examinations performed is below the target value, this is considered “under-performance”, while a higher value is referred to as “over-performance”.

The multi-professional work team determined the timeframe for the competency assessment, in particular the four-month interval chosen to evaluate the examinations performed by each radiographer for each macro-group and compare them with the defined target areas. When determining this timeframe, careful attention was paid to possible distortions that could arise due to the absence of radiographers due to illness or holidays. Recognizing that shorter assessment periods could increase the impact of bias, particularly in the summer months when radiographers are more likely to be on holiday, the team opted for a four-month period to ensure a more balanced and comprehensive assessment. In addition, the competency assessment took into account the radiographer’s professional experience gained over the last three years. If the number of examinations performed exceeded a threshold (set by the multi-professional work team) of 3000 examinations performed in the previous three years based on the date of data extraction, the radiographer is automatically recognized as competent for macro-groups that are not considered complex for performing examinations. These aspects related to the competency assessment timeframe are customizable and play a crucial role within the SRH tool. They have been the subject of optimization and multi-professional discussions.

**Figure 2:**
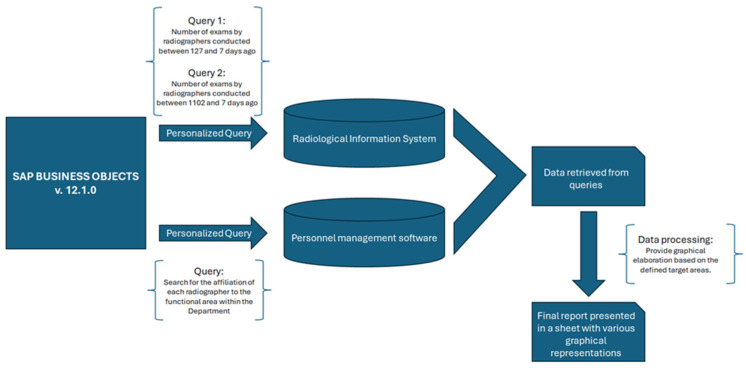
Diagram illustrating the methodology, software, and queries used for developing the SRH tool.

The development of the SRH tool involved the use of SAP Business Objects. This process involved the execution of targeted queries in the RIS and personnel management software systems, as shown in Figure 2. Specifically, two different queries were created to extract information from the RIS about the radiographers’ work experiences. These queries assessed recent activity by quantifying the number of examinations performed in the last 4 months for each macro-group and past experience by evaluating the number of examinations performed in the previous 3 years. In addition, a specific query was created to obtain information about each radiographer’s work area from the Careggi hospital’s personnel management software. These data are crucial for the transmission of the report on the radiographers who fall within the area of responsibility of the respective coordinator.

Once the relevant data were retrieved from these software systems, they were merged together and analyzed graphically based on the target areas identified by the focus group.

Subsequently, the analyzed data were compiled into a final report, which was presented in an Excel file featuring various graphical representations. These graphical representations provided a comprehensive overview of the findings, facilitating easier interpretation and decision-making processes. 

The final report is compiled and dispatched weekly to the coordinator, enabling dynamic adjustments to daily work shifts. This proactive approach aids in mitigating instances of both underperformance and overperformance among radiographers, ensuring a balanced workload distribution and the optimal utilization of resources.

To enhance the understanding of radiographer performance, the final report includes visual aids alongside textual information. These visuals are crucial for presenting data clearly and concisely. The report, formatted as an Excel file, uses various graphical elements to illustrate the competencies of each radiographer, highlighting both individual and collective performance metrics.

**Figure 3:**
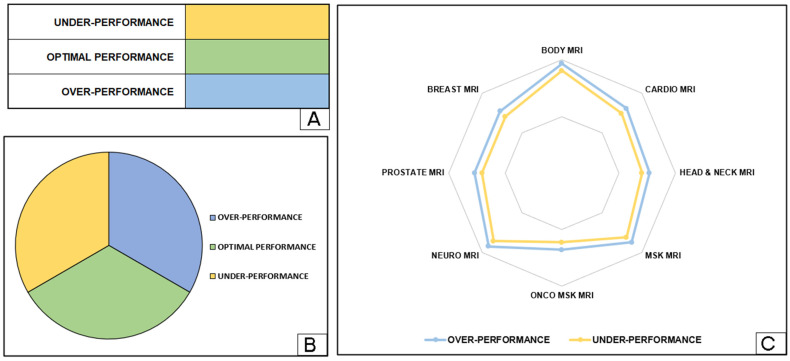
The method for visualising the competences for each radiographer is presented in the final report produced by the SRH tool. In particular, the visualization of the table method (**A**), the pie chart (**B**) and the radar chart (**C**) is presented.

Figure 3 demonstrates how the graphical representations of each radiographer’s competencies are reported in the final document. This figure represents the style of visualization used for the report generated and sent to coordinators to optimize daily work shifts. In detail:

- Figure 3A shows a table listing the competencies for each macro-group assigned to radiographers based on defined target areas. To improve readability and interpretation, different colors indicate specific target areas within the competencies.
- Figure 3B includes a comprehensive pie chart providing a holistic overview of the competencies across all macro-groups for all radiographers. This diagram offers an e-summary of the distribution of skills and expertise within the radiography team, facilitating informed decision making and resource allocation for coordinators.
- Figure 3C features a radar chart that graphically depicts the competencies of each radiographer for the different MRI macro-groups. This visualization technique provides a comprehensive overview of the professionals’ competencies and areas for improvement in their respective fields. Optimal performance is defined as the area between two lines indicating underperformance and overperformance.

Incorporating these visual elements into the final report not only enhances the clarity and impact of the data but also aids coordinators in making informed decisions regarding shift adjustments and resource allocation. The graphical representations allow for a quick and intuitive understanding of performance metrics, ensuring that radiographers are effectively utilized and that their workload is balanced. This detailed and visually enriched reporting system underscores the importance of continual performance monitoring and adjustment in the radiography department. By providing a clear and comprehensive overview of individual and collective competencies, the final report supports a proactive approach to workforce management. This ensures that each radiographer’s skills are optimally utilized, contributing to overall efficiency and improved patient care outcomes.

The graphical representations included in the final report serve to enhance the textual data by providing stakeholders with a more detailed and thorough analysis of radiographer performance. These visuals offer a comprehensive overview of key metrics and trends, offering valuable insights into areas of strength and improvement.

**Figure 4:**
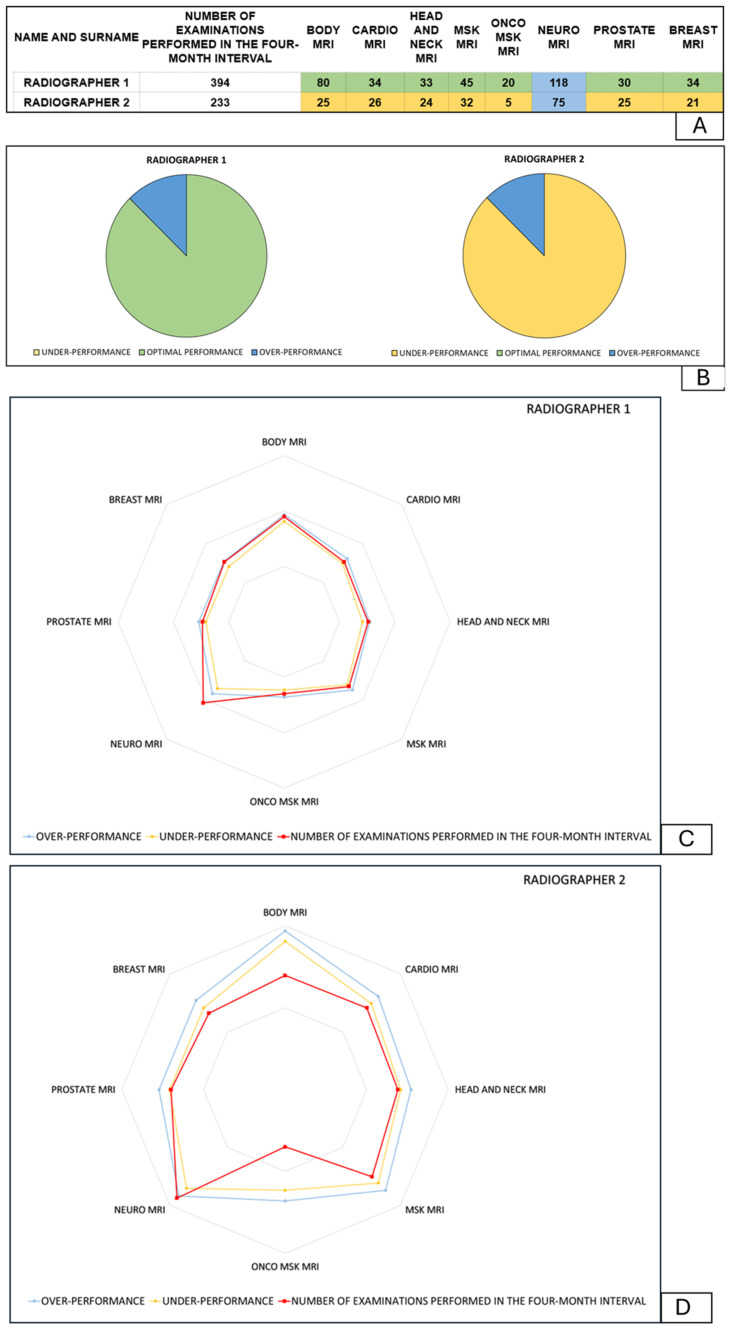
Comparison of the competences of radiographer 1 and radiographer 2 in MRI examinations with visualization of the table method (**A**), the pie chart (**B**), and the radar chart (**C**,**D**).

Figure 4 shows a visualization of the report provided in clinical practice to the coordinator with data on MRI examinations performed by two radiographers. As shown in Figure 4A–C, radiographer 1 shows optimal competences in performing MRI examinations, while radiographer 2 shows under performance in all major MRI categories except neuro-MRI examinations. The difference between the two radiographers is clearly emphasized both numerically in the table in Figure 4A and graphically in the pie chart and radar chart in Figure 4B and Figure 4C, respectively.

**Figure 5:**
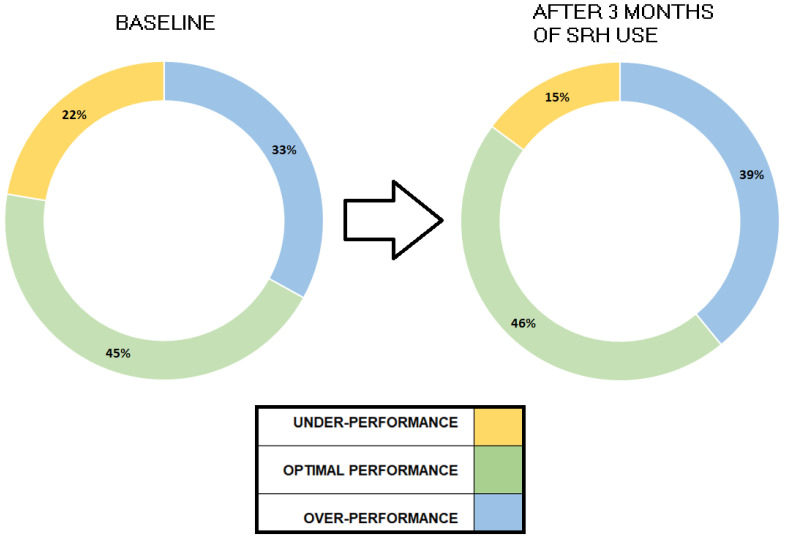
Preliminary data from the application of the SRH tool in clinical practice for MRI examinations.

Furthermore, the development and implementation of the SRH tool was accomplished without the need for additional resources, as no new software was purchased. The staff involved in the focus group and multi-professional working team were highly skilled professionals from the Department of Allied Health Professions or other Departments within Careggi Hospital, ensuring a high level of expertise and collaboration throughout the process. This collaborative approach allowed for a holistic and effective evaluation of radiographer performance, ultimately leading to a more informed and strategic decision-making process for all stakeholders involved. After a trial phase of implementing the SRH tool into clinical practice, preliminary data were collected and analyzed. In this initial trial phase, the tool was implemented in clinical practice by two coordinators to optimize the work shifts of 35 radiographers for MRI examinations. The analysis of these data indicated a significant improvement in radiographers’ competencies. After three months of using the tool, a 7% reduction in radiographer underperformance in MRI examinations was observed (see Figure 5). This tool therefore enabled a reduction in underperformance from 22% at baseline to 15% after three months of using the SRH tool. In particular, this innovative tool allows for the optimization of work shift scheduling by aligning it with the specific competencies and skills of the staff members. The successful outcomes of this approach were showcased during a focus group session held by the Allied Health Professions Department at Careggi Hospital. This initiative forms part of a broader strategy aimed at fostering continuous improvement in the management of human resources within the healthcare facility.

### 3.2. Feedback from the Training Event before the SRH Tool Release

#### 3.2.1. Insights into the Participants to the Training Event

An important outcome of this study is the creation of an anonymous electronic survey at the end of the focus group sessions that was facilitated by the CAWI tool. This survey was designed to collect structured feedback on the use of the SRH tool in clinical practice. It is noteworthy that the survey was completed quickly. Overall, 32 coordinators from 11 different professional profiles participated in the departmental meeting. In total, 29 coordinators answered to the questionnaire. The response rate was 90.6% (*n* = 29). Participants took an average of 285.3 ± 140 s to open and complete it, with the entire process not exceeding 550 s. Once the survey was opened, all participants readily provided their responses. The two tables (Table 2 and Figure S4) provide details of the overall sample of healthcare professionals who responded to the survey (Table 2) and details of the professional profiles of questionnaire respondents (Figure S4). The first table provides a comprehensive overview of the entire sample surveyed, while the second table looks specifically at the professional profiles of the individual respondents. The mean age of responders was 50.8 (±9.5), with a higher prevalence of being female (68%).

Most of the participants were radiographers (*n* = 9, 31.0%), as can be seen from the Supplementary Materials (Figure S4), which shows the healthcare disciplines participating in the questionnaire.

#### 3.2.2. Feedback and Findings from Single-Choice and Open-Ended Questions 

The analysis of the completed questionnaires shows that more than 85% of the respondents found the use of the tool presented to be easy to understand (*n* = 25, 86.2%), but nevertheless considered specific training to be necessary for the use of this tool in clinical practice (*n* = 25, 86.2%). Regarding the use of the tool, more than two-thirds of the respondents thought that it could provide a valuable decision-making aid in the organization of work shifts and that the use of the processed data could support competence management (*n* = 20, 69% and *n* = 21, 72.4%, respectively). Furthermore, according to the majority of respondents, the use of this tool in clinical practice could play an important role in minimizing clinical risks and lead to a reduction in errors and adverse events (*n* = 20, 69%). A total of 75.8% of respondents (*n* = 22) expressed their belief that this tool can be implemented in their work environment. From the responses obtained, it emerges that the SRH tool finds applicability in clinical practice across almost all the different healthcare profiles (*n* = 10, 83%) affiliated with the Department of Allied Health Professions. In cases where respondents answered negatively, they were asked to provide reasons through an open-ended question. Analysis of the responses to the open-ended question revealed that:

- In the field of cardiovascular perfusion, the utilization of the SRH tool in clinical practice may be restricted due to the necessity of having all colleagues present for both emergency and routine activities.
- A total 71% of the medical laboratory technician coordinators are of the opinion that this tool cannot be implemented in this form but that appropriate modifications would be necessary, as there is not always a match between medical laboratory technicians and performance and not all of their activities are measurable.

Furthermore, the vast majority of respondents find it easy to identify “target ranges” for the competency analysis (*n* = 25, 85.2%). Most respondents would be willing to recommend the use of this tool to other organizations and/or companies in the healthcare sector (*n* = 22, 75.9%).

#### 3.2.3. Feedback and Findings from Likert-Scale Questions

**Figure 6:**
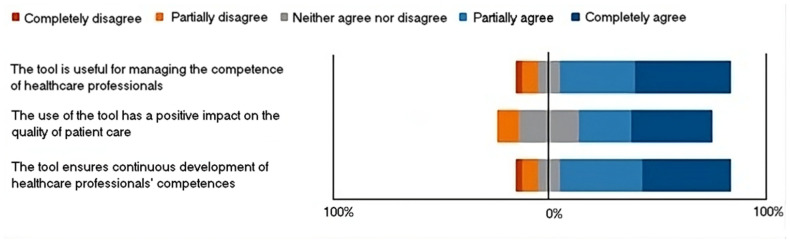
Representation scheme of the answer to the Likert sentences.

In the assessment, individually graded and Likert responses were employed, with a scale ranging from a maximum score of 5 to a minimum of 1 (Figure 6). An average score surpassing 3.0 = 1 + 5/2 signified a positive evaluation, with a higher score approaching 5 indicating a more favorable response. Conversely, a score falling below 3.0 signaled a negative evaluation, with a score approaching 1 indicating a more critical stance. 

The butterfly diagrams clearly show that the tail is only minimally below 0% for all options. This observation indicates a consistently high level of favorable evaluation for each choice presented with regard to the use of this tool in clinical practice.

Details:

Within the Likert scale associated with the set of options for “the tool is useful for managing the competence of healthcare professionals”, the most favored choice was “Completely agree” (*n* = 13), garnering the highest average rating of 4.1 (STD ± 1.3).

Within the Likert scale associated with the set of options for “the use of the tool has a positive impact on the quality of patient care”, the most favored choice was “Completely agree” (*n* = 11), garnering the highest average rating of 3.9 (STD ± 1.2).

Within the Likert scale associated with the set of options for “the tool ensures continuous development of healthcare professionals’ competences”, the most favored choice was “Completely agree” (*n* = 12), garnering the highest average rating of 4.1 (STD ± 1.3).

#### 3.2.4. Feedback from Qualitative Analysis

After completing the survey and sharing the results, an interdisciplinary and multiprofessional discussion was held on the implementation of the SRH tool. Based on this discussion, a brief summary was prepared, as shown in Table 3, outlining the most discussed topics and the “unbiased feedback” associated with these topics from potential SRH tool users.

### 3.3. Comprehensive SRH Tool Release, Maintenance, Monitoring, and Transfer Plan

#### 3.3.1. SRH Tool Release

The software is developed with meticulous attention to cybersecurity and software quality standards, ensuring the highest levels of security and reliability. Rigorous quality assurance processes, including comprehensive testing and code reviews, guarantee its reliability, scalability, and performance. Its seamless integration capability with robust and well-established databases streamlines implementation and enhances data integrity and accessibility. By prioritizing cybersecurity, software quality, and robust database integration, the software offers a resilient and future-proof solution for diverse application scenarios. Aligned with distributed quality concepts in hospital and care, it meets the specific needs of healthcare professionals within the public healthcare framework, facilitating ongoing quality assurance efforts and promoting collaboration among stakeholders. Overall, its adherence to distributed quality concepts enhances healthcare service delivery within the public healthcare system.

#### 3.3.2. SRH Tool Maintenance and Monitoring

The maintenance and monitoring of the SRH Tool will be significantly enhanced through two pivotal strategies:

- Implementation of Quality Control and Monitoring Procedures: This strategy involves establishing robust protocols and procedures to ensure the seamless functionality and reliability of the SRH tool. By adhering to stringent quality control measures, potential issues can be pre-emptively identified and addressed. These procedures will align closely with organizational standards, ensuring consistency and adherence to best practices. Meticulous monitoring at every stage of the SRH tool’s lifecycle will proactively safeguard against disruptions and optimize performance.
- Application of Key Performance Indicators (KPIs) Based on the Scientific Literature: Monitoring efforts will be fortified by the application of KPIs selected based on extensive research from the scientific literature. Drawing upon insights from peer-reviewed studies and industry best practices, meaningful indicators of the SRH tool’s effectiveness and efficiency will be identified and prioritized. Aligning monitoring efforts with evidence-based metrics will provide comprehensive insights into strengths and areas for improvement. This data-driven approach will empower informed decision making and drive continuous enhancements to the SRH tool’s functionality and performance.

By focusing on these two cornerstone principles, a robust framework for the maintenance and monitoring of the SRH tool will be established, fostering a culture of excellence and innovation within the organization.

##### Implementation of Quality Control and Monitoring Procedures for the SRH Tool 

In the rapidly evolving landscape of healthcare, the introduction of sophisticated tools like the SRH tool marks a significant advancement. However, its successful implementation requires more than just technology deployment; it necessitates a comprehensive strategy encompassing maintenance, monitoring, and feedback integration to ensure continual enhancement. At the core of this strategy are maintenance procedures, which form the bedrock of reliability for the SRH tool. Robust maintenance protocols guarantee uninterrupted functionality; preempting potential disruptions and engaging diverse stakeholders further strengthens reliability through a distributed maintenance approach, fostering collaboration and expediting issue resolution. Central to this multifaceted approach is the monitoring framework, integrating distributed quality indicators to comprehensively evaluate the SRH tool’s effectiveness and efficiency. Real-time data collection and analysis provide actionable insights for proactive refinement, while quality assurance mechanisms uphold uncompromising standards, fostering collective dedication to excellence. An essential aspect of this process is the seamless integration of feedback loops, facilitating continuous improvement based on insights from users and stakeholders. Incorporating dedicated feedback questionnaires refines functionality and enhances user experience, ensuring alignment with evolving needs. Driving this strategy is an agile implementation approach, ensuring that feedback is actively analyzed and acted upon. Transparent communication and heightened accountability foster a culture of responsiveness and continuous improvement, ultimately optimizing the SRH tool’s efficacy and user experience, guiding healthcare organizations towards excellence in service provision and patient care amidst dynamic healthcare landscapes.

##### Application of Key Performance Indicators Used for Fostering Effective Communication and Leadership Using the SRH Tool

Creating a communication chain that facilitates progress and empowers internal leadership based on Key Performance Indicators (KPIs) in a healthcare setting, particularly within the context of utilizing the SRH tool, involves leveraging the best practices outlined in the existing literature. By selecting relevant KPIs [22,23,24,25,26,27,28,29,30,31], a structured approach can be developed to guide the implementation and continuous improvement of the SRH tool.

Establishing clear objectives and KPIs is the first step. This involves defining the goals of the SRH tool in alignment with the healthcare organization’s overall objectives, such as improving quality and efficiency in radiology services [22]. Selecting relevant KPIs, such as report turnaround time, error rates, and user satisfaction, ensures accurate measurement of performance, quality, and outcomes [23].

Developing transparent communication channels is crucial for ensuring information flows freely. Establishing multiple communication channels, such as team meetings, digital platforms, and email updates, helps maintain regular updates on KPI progress and tool performance, fostering a culture of transparency [24,25]. 

Fostering a collaborative environment through regular team meetings and feedback sessions encourages open feedback and collaborative problem solving. Cross-functional teams, including members from different departments, provide diverse perspectives on the KPI results and improvement strategies [26,27]. 

Implementing leadership development programs is essential for the effective use of KPIs. Training programs focusing on leadership skills, effective communication, and data-driven decision making help leaders interpret KPIs and make informed decisions. Mentorship programs, where experienced leaders guide team members, also play a key role in understanding and utilizing KPIs [28,29]. 

Empowering team members by delegating responsibilities related to KPI tracking and reporting fosters ownership and accountability. Providing access to KPI data and analysis tools promotes transparency and trust within the team [30,31]. 

Recognizing and rewarding achievements based on KPI progress boosts morale and motivates the team. Regularly celebrating successes and implementing incentive programs for meeting or exceeding KPI targets are effective strategies [22,23]. 

Finally, continuous improvement and adaptation are vital. Establishing a feedback loop allows team members to suggest improvements based on KPI outcomes. Being agile and willing to adapt KPIs and strategies based on performance data and changing organizational goals ensures ongoing relevance and effectiveness [24,25]. Table 4 reports the selected references and relevant KPIs. Table 5 provides a comprehensive breakdown of the areas of intervention, accompanied by related studies, showcasing the application of key performance indicators utilized to enhance effective communication and leadership through the utilization of the SRH tool.

The adoption of this structured approach based on the KPIs can ensure the SRH tool is effectively utilized and integrated, promoting a responsive and dynamic environment that prioritizes continuous improvement and effective leadership.

#### 3.3.3. SRH Transfer Plan

The SRH tool, originally designed for radiographers and localized within the Careggi Hospital unit, was introduced at a formative event prior to its release. This introduction was followed by an initial Computer-Assisted Web Interview (CAWI) to provide initial guidance. The questionnaire served as a mechanism for approval and initial feedback. During the software’s deployment phases, targeted questionnaires will be utilized as monitoring and feedback tools, and they will also play a critical role during the transfer phase. Transferring the software to other professions represents a broader and more complex challenge. The CAWI conducted during the formative event indicated that the tool’s exportability to all professions is not guaranteed. It highlighted the necessity of initiating a significant dialogue with involved stakeholders, particularly professional associations and the professionals themselves, including those in managerial roles.

There are 19 healthcare professions involved in the transfer of the SRH tool, including radiographers, for whom the tool was originally designed. Each of these healthcare professions has category associations representing their respective professional orders.

The first is The National Federation of Orders of Medical Radiology Technicians and Technical Health Professions of Rehabilitation and Prevention, which represents 18 of the 19 healthcare professions involved in the transfer of the tool (see the web site of the association at: https://www.tsrm-pstrp.org/, accessed on 10 June 2024). The other is The National Federation of the Order of Physiotherapists, which represents the fifteenth profession involved in the department (see the website of the association at: https://www.fnofi.it/, accessed on 10 June 2024).

The transfer process will require dialogue and strong interaction with these associations to enable the creation of adapted and targeted versions of the SRH tool for each of these professions. The transition of the SRH tool to various professions hinges on the creation of targeted and specialized questionnaires for each profession. This will process involve extensive collaboration with professional associations, stakeholders, and experts in social sciences and communication throughout the design, submission, and data analysis phases of the surveys. Initially, survey design is crucial. This involves engaging with professional associations and stakeholders to ensure the questionnaires are relevant and comprehensive. Experts in social sciences and communication will play a key role in developing these questionnaires, ensuring they are tailored to address the specific needs and contexts of each profession. Once the surveys are designed, the next step is to assess the feasibility of transferring the SRH tool to the targeted profession. This involves evaluating whether the tool can be effectively adapted to meet the unique requirements of the profession. If the transfer is deemed feasible, the process moves forward; if not, the reasons are documented and further steps for that profession are halted. For professions where feasibility is confirmed, both the general and specific requirements will be gathered. This step ensures that all necessary aspects are covered, including unique challenges and needs specific to the profession. Next, strategic observations relevant to the implementation process will be collected and documented to provide a comprehensive understanding of the context. Following this, the questionnaires will be distributed to the relevant professionals. Ensuring anonymity in submissions is essential to encourage honest and candid responses, which leads to more reliable data. The collected data will then be then systematically analyzed to extract key insights and determine actionable steps.

The integration phase involves incorporating the gathered requirements and strategic observations into the SRH tool. This customization ensures that the tool is specifically adapted to fit the unique context of each profession. The final step is establishing ongoing feedback mechanisms. These mechanisms are crucial for continuous improvement, allowing the SRH tool to be regularly updated and refined based on user feedback and evolving requirements. This structured and detailed approach ensures that the SRH tool is effectively adapted and implemented across various healthcare professions. By doing so, it fosters a comprehensive and responsive healthcare environment that can better meet the diverse needs of different professional contexts.

## 4. Discussion

### 4.1. Highlights from the Study

The work delved into the emerging topic of optimizing work shifts through a skills-oriented lens. Specifically, it centered on the vital aspect of skill retention within healthcare professions, with a particular focus on the role of radiographers. This subject holds significant importance within the healthcare sector, not merely for upholding high standards of service delivery and ensuring that professionals have a suitable and efficient workload but also for nurturing the professional development of individuals through mentoring initiatives. Furthermore, it is crucial for the overall effectiveness and efficiency of hospital systems, facilitating better service delivery and cost management.

The study offers several noteworthy contributions. 

Firstly, it introduces a computerized and customized tool, the SRH tool, designed for monitoring the maintenance of professional skills. This tool stands out for being developed at no additional cost for software products, as it leverages existing software and databases routinely used for other activities. Moreover, it is designed to be easily exportable, both in its current form and as a model for computerized relationships and queries. While initially tailored to a specific healthcare profession, it is adaptable for use across various healthcare roles, albeit with some identified limitations.

Additionally, the study highlights the ability of the SRH tool to generate weekly reports that can be presented to activity coordinators. These reports not only aid in managing activities but also contribute to the judicious allocation of resources, minimizing risks for both the workforce and the patients they serve. Ultimately, the tool helps ensure that workloads and assigned activities are appropriately matched, optimizing overall efficiency. 

The second significant contribution involves the creation of a CAWI tool tailored for evaluating the SRH tool’s acceptance after a training event. This CAWI instrument, designed to assess the SRH tool or similar computerized tools, underwent testing with a cohort of coordinators. Through this quantitative and tangible assessment, it was revealed that there was both a robust acceptance of the proposed computerized solution for skill maintenance and an expressed interest in its potential application across other healthcare professions, albeit with caveats. Within the open-ended responses, the CAWI tool functioned akin to a virtual focus group, elucidating key insights and offering valuable suggestions. Noteworthy recommendations included:

- Recognition of the impracticality of extending the model/product to medical laboratory technicians and cardiovascular perfusionists due to the intricacies of their shift scheduling.
- The imperative need for specialized training integrated into the curriculum of Italian university degree programs.
- The usefulness in the hands of the relevant professional scientific society/associations. This comprehensive evaluation mechanism not only provided concrete feedback on the SRH tool’s viability but also yielded actionable insights for refining its implementation and potential expansion into diverse healthcare sectors.

The third significant contribution is the first outcome after a first implementation in clinical practice by two coordinators to optimize the work shifts of 35 radiographers for MRI examinations. In fact, after three months of using the tool, a 7% reduction in radiographer underperformance in MRI examinations was observed. This showcased the potential of the tool in clinical routine practice in relation to the competence monitoring and improvement of professionals in the health domain.

The fourth significant contribution Is the definition of the roadmap for the SRH tool’s maintenance, monitoring, and transferring. The importance of the SRH tool’s maintenance, monitoring, and transfer plan lies in its meticulous approach to ensuring reliability, optimizing performance, and adapting to diverse healthcare needs. By prioritizing cybersecurity and software quality standards, the plan guarantees robustness and scalability. Through quality control procedures and evidence-based KPIs, ongoing enhancement and effectiveness are ensured. The transfer plan, facilitated by targeted questionnaires and collaboration with professional associations, enables seamless adaptation to various healthcare professions, fostering a responsive and inclusive healthcare environment.

### 4.2. Importance and Location of the Study

To understand the global need of this study, we explored international research using specific composite keywords (Box S1, positions 1–3) and included the findings in the construction of the study’s introductory hypotheses. This examination revealed a significant increase in interest in healthcare professionals’ workloads, competences, and performance and the related relationships, particularly over the past five years during the global pandemic, strongly motivating this study (Figures S1–S3 in the Supplementary Materials). Indeed, the trends highlighted in the premises of this study underscore the crucial role these professionals have played during the pandemic and the growing focus on optimizing their work dynamics.

Also, a literature review conducted to define the methodology for determining the required competence levels for radiographers highlighted the importance of clinical experience in achieving high clinical competence. This finding aligns with the study by Yezengaw et al. (2023) [17], which emphasized the significance of clinical practice competence among healthcare students in Northwest Ethiopia. Additionally, Budoff et al. (2005) [18] emphasized that understanding clinical competence in cardiac imaging requires considering factors like the number and variety of examinations performed.

Our tool for optimizing work shifts for healthcare professionals, particularly radiographers, directly aligns with the findings of the literature review on determining competence levels. Specifically, the tool takes into account the crucial role of clinical experience in achieving and maintaining clinical competence, as emphasized in the literature. By optimizing work shifts to provide adequate opportunities for clinical practice and exposure, our tool supports radiographers in acquiring and honing the necessary skills and competencies highlighted in the literature. Moreover, our tool considers specific requirements identified in the literature, such as the number of examinations needed to achieve certain levels of clinical competence. By ensuring that radiographers have sufficient workloads and exposure to patient cases, our tool facilitates the attainment of these competence levels. In essence, our tool for optimizing work shifts for radiographers directly addresses the key findings of the literature review by providing a practical solution to support the development and maintenance of clinical competence through tailored shift scheduling. As in any other field, competences, workload, and performance are closely interconnected. The discourse on workload, competence, and performance among healthcare professionals spans multiple dimensions, drawing insights from a diverse array of studies. The study by Portoghese et al. (2014) [32] sheds light on the intricate interplay between burnout, workload, and job control among healthcare workers. It underscores how high workload levels can contribute to burnout, ultimately affecting job performance and satisfaction. Building upon this, Kovacs and Lagarde’s (2022) [33] research explores the impact of high workload on healthcare quality, revealing potential repercussions on the quality of care provided. Moreover, Rostami et al. (2021) [34] delve into the relationship between mental workload, job satisfaction, and job control among healthcare workers. They highlight the moderating role of job control in mitigating the adverse effects of mental workload on job satisfaction and, consequently, on performance. Furthermore, Jose et al. (2022) [35] discuss the significance of professional competence in the implementation of healthcare technologies. Competent healthcare professionals are better equipped to leverage technological advancements effectively, potentially enhancing performance and patient outcomes. Yaqoob et al. [36] conducted a systematic review focusing on healthcare professionals’ core competency instruments. They examined various tools and methods, in line with our study, used to assess the core competencies of healthcare professionals.

Additionally, studies such as those by Jarva et al. (2024) [37] and Krijgsheld et al. (2022) [38] delve into the digital health competence and overall job performance of healthcare professionals, respectively. They underscore the importance of staying abreast of digital advancements and maintaining competence to ensure optimal performance in healthcare delivery. Rana et al. [39] investigated job satisfaction, performance appraisal, reinforcement, and job tasks among medical healthcare professionals during the COVID-19 pandemic, while the study by Crawshaw et al. [40] was indirectly related to the performance of healthcare professionals by identifying the behavior change techniques used in interventions targeting practice change. By understanding which techniques are effective in prompting changes in healthcare practices, organizations can implement strategies that may ultimately enhance the performance of healthcare professionals. Improving adherence to evidence-based practices through effective interventions can lead to better outcomes for both healthcare providers and patients, potentially reflecting positively on performance metrics.

In light of these findings, it becomes evident that workload, competence, and performance are intricately linked within the healthcare domain. Managing workload levels, fostering professional competence, and ensuring job satisfaction are all critical factors that can ultimately influence the quality of care delivered by healthcare professionals. Thus, a holistic approach that addresses these interconnected factors is essential for promoting optimal performance and enhancing healthcare outcomes. Examining from three interconnected perspectives—workload, competence, and performance—the complementary value of our study becomes evident.

First and foremost, our study focuses on optimizing work shifts for healthcare professionals, recognizing the crucial role that workload plays in their ability to effectively carry out their duties. By directly addressing workload management, we are able to directly influence healthcare professionals’ capacity to perform their tasks efficiently and effectively.

Secondly, our study also considers the importance of healthcare professionals’ competence, particularly radiographers. We acknowledge that high competence is fundamental to ensuring the quality of care and patient safety. Consequently, we design our work shifts to allow healthcare professionals to maintain and develop their professional skills over time.

Finally, optimizing work shifts and supporting healthcare professionals’ competence directly translate into improved overall performance of the healthcare organization. By reducing the risk of burnout and workload-related fatigue and ensuring that healthcare professionals are adequately trained and competent, we can enhance the quality and efficiency of healthcare services provided. Overall, our study fits into a cycle of continuous improvement, where optimizing work shifts supports healthcare professionals’ competence, which in turn results in improved organizational performance. These three dimensions are closely intertwined and mutually reinforce each other, highlighting the complementary and crucial value of our work in healthcare management.

Our study should also be considered within a broader context that encompasses the multifaceted dynamics of healthcare systems and professional development. In addition to examining the implications of our findings, it is essential to recognize the pivotal roles played by scientific associations in shaping the landscape of healthcare professions. These associations serve as crucial platforms for knowledge exchange, professional networking, and advocacy, thereby influencing the evolution of healthcare practices and policies. Moreover, the functional organization of hospitals and healthcare institutions significantly impacts the implementation and utilization of tools such as SRH and CAWI. Effective integration of these tools into existing workflows requires alignment with organizational structures and processes. Furthermore, understanding the emerging roles and competencies within healthcare professions, including those related to teleradiology (Chandramohan et al. [41]), home radiology (Lepri et al. [42]), and other innovative healthcare delivery models, is essential for ensuring that these tools are utilized to their full potential. As new technologies continue to reshape healthcare delivery, it becomes imperative to adapt training programs and job descriptions to equip healthcare professionals with the necessary skills and knowledge to navigate these changes effectively. The establishment of dedicated departments or units focused on healthcare professions, as exemplified in our case, can facilitate centralized management and coordination of initiatives related to professional development and tool implementation. By centralizing resources and expertise, these departments can streamline processes and ensure consistency in training and competency assessment across different healthcare settings. Looking ahead, the increasing prominence of technologies such as artificial intelligence (AI) presents both opportunities and challenges for healthcare professionals (Giansanti et al. [43]). While AI has the potential to enhance diagnostic accuracy, streamline workflows, and improve patient outcomes, its integration into clinical practice requires careful consideration of ethical, legal, and regulatory frameworks. Furthermore, tools like SRH may evolve to incorporate AI-driven algorithms for optimization, clustering, and other advanced functionalities, thereby enhancing their utility and effectiveness in supporting healthcare professionals. In summary, our study underscores the interconnected nature of various factors influencing healthcare workforce management and professional development. By embracing innovative healthcare delivery models and integrating emerging technologies, such as teleradiology and home radiology services, healthcare systems can enhance the quality and efficiency of patient care. The proposed SRH toll can play a pivotal role in this direction by offering insights into workload optimization, competency assessment, and performance enhancement among healthcare professionals. Through centralized management and ongoing training initiatives, organizations can adapt to changing roles and technologies, ultimately improving patient care outcomes and meeting the demands of modern healthcare delivery.

## 5. Work in Progress

Several developments have been outlined for advancing this initiative concerning the development, dissemination, and implementation of the SRH tool. The SRH tool will be deployed and shared within the Pisa University Hospital network, as there is an active collaboration between the Department of Allied Health Professions of Careggi Hospital and Pisa University Hospital. Currently, colleagues from the Department of Allied Health Professions at Pisa University Hospital have already carried out a multidimensional analysis using the health technology assessment method for preventive evaluation prior to the introduction of the new model. The next step will be to adapt the SRH tool into their context.

After an initial 6-month trial period at the Careggi hospital, an initial report will be compiled to analyze outcomes, while concurrently gauging user satisfaction and acceptance through targeted assessments.

In addition to its primary objectives, the SRH tool indirectly facilitates the advancement of personalized medicine by aligning the competence data of healthcare professionals with patient data. This alignment enables healthcare providers to offer tailored treatment plans based not only on patient needs but also on the specific skills and expertise of the professionals involved. Furthermore, the integration of AI algorithms holds significant potential in this regard. AI could analyze the vast amounts of data collected by the SRH tools, identifying patterns and correlations that might not be immediately apparent to human analysts. This analytical capability could lead to the development of personalized medicine approaches that are more precise and effective.

By leveraging AI, healthcare institutions can conduct in-depth investigations into the correlations between healthcare provider competence, patient outcomes, and treatment efficacy. AI algorithms can sift through vast datasets to identify trends and insights that could inform decision-making processes. This synergy between technology and healthcare practice could revolutionize the delivery of personalized medicine, ensuring that treatments are not only tailored to individual patient needs but also optimized based on the expertise of the healthcare professionals involved. Another important activity to be implemented is the SRH tool’s maintenance, monitoring, and transfer plan. Its significance lies in its meticulous approach to ensuring reliability, optimizing performance, and adapting to diverse healthcare needs. By prioritizing cybersecurity and software quality standards, the release plan guarantees robustness and scalability. Through the implementation of quality control procedures and the application of evidence-based KPIs, the maintenance and monitoring plan ensures ongoing enhancement and effectiveness. Meanwhile, the transfer plan, facilitated by targeted questionnaires and collaboration with professional associations, enables seamless adaptation to various healthcare professions, fostering a responsive and inclusive healthcare environment.

## 6. Takeaway Message

The study emphasizes the importance of optimizing work shifts in healthcare through a skills-oriented approach, particularly focusing on radiographers. The development of the SRH tool offers practical solutions for skill monitoring and assessment, ultimately improving healthcare professionals’ competence, workload management, and organizational performance. Integration with AI algorithms holds promise for further enhancement.

## 7. Conclusions

In conclusion, this study sheds light on the critical aspect of optimizing work shifts in healthcare, with a particular focus on skill retention among radiographers. The development of the SRH tool offers practical solutions for monitoring skills and evaluating their efficacy, thereby contributing to enhanced competence, workload management, and organizational performance. Key takeaways include:

- Introduction of the SRH tool, a cost-effective solution leveraging existing software, tailored for radiographers.
- Highlighting the SRH tool’s ability to generate weekly reports aiding in activity management and resource allocation.
- Creation of the CAWI tool for evaluating the acceptance of the SRH tool, providing valuable feedback and insights after the training event before its release, also useful for defining a roadmap for transferring it to professions other than radiographers.
- Initial outcomes from the first implementation in clinical practice by two coordinators to optimize the work shifts of 35 radiographers for MRI examinations. After three months of tool usage, a 7% reduction in radiographer underperformance in MRI examinations was observed. This outcome on the profession of radiographers showcases the tool’s potential in routine clinical practice for monitoring competence and improving professionals in the healthcare domain.
- Definition of a roadmap for monitoring-and- maintenance and, specifically, transferring the SRH tool conducting feasibility investigations and making adaptations tailored to various healthcare professionals, with the participation of relevant scientific associations. This approach will enable the release of a customized version of the tool.

Moving forward, the deployment of the SRH tool within the Pisa hospital network and potential integration with AI algorithms are anticipated enhancements for further optimizing its utility and effectiveness.

## Figures and Tables

**Table 1:** Key elements investigated in the survey.

Key Elements
Impact of the SRH tool in clinical practice
Applicability across various healthcare profiles
Value in decision making
Competence analysis
Simple understanding and training
Improvement in clinical risk management

**Table 2:** Sample of healthcare professionals interviewed using the electronic survey.

Participants	Males/Females	Mean Age ± St.d.	Min/Max Age
29	9/20	50.8 ± 9.5	32/64

**Table 3:** Summary of Key Discussion Topics and Unbiased Feedback from Potential SRH Tool Users.

Key Discussion Topics	Unbiased Feedback	Responses Provided to Feedback
Delineation of “target areas”	“Without guidelines, how can the focus group outline the target areas?”	The focus group consists of experienced healthcare professionals who can identify specific areas of focus through a comprehensive evaluation.
	“Are target areas customizable even after importing SRH tools into clinical practice?”	These target areas can be adjusted as needed, and the SRH tool will update evaluations accordingly based on the new target areas.
Reporting of examination performed by individual healthcare professional	“We cannot implement the SRH tool because the activities and performance metrics of these professionals cannot be measured into a software platform.”	The functionality of the SRH tool is inevitably compromised if professionals are unable to provide a report of the examinations they have performed.
	“In my clinical practice, examinations are usually performed and recorded by two healthcare professionals. Would the SRH tool have problems extracting the data in this case?”	No, the SRH tool is able to retrieve and process the data by defining specific queries, taking into account that there are multiple examiners (even more than one).
The use of the SRH tool for optimizing work shift	“The SRH tool does not allow automatic management of emergency situations (e.g., employees who do not report their absence unannounced).”	The SRH tool was not developed to manage critical situations independently, but it helps the coordinator to optimize the work shift based on the skills of the individual healthcare professionals. In the event of a sudden absence, it helps the coordinator to find a replacement.
SRH tool improves decision-making processes	“The most common criticism of a coordinator relates to subjectivity when planning work shifts. Can the SRH tool help to make the management of work shifts more objective?”	Yes. By providing objective, data-driven insights, the SRH tool enables coordinators to make management decisions with confidence and accuracy.

**Table 4:** The selected references and the relevant KPIs.

Reference	Relevant KPIs
Abujudeh et al., 2010 [22]	Report turnaround time, quality and efficiency metrics
Nelson et al., 2022 [23]	Error rates, user satisfaction
DiCostanzo et al., 2022 [24]	Frequency and quality of communication updates
European Society of Radiology, 2020 [25]	Transparency and engagement levels
Schultz et al., 2016 [26]	Team meeting effectiveness, feedback quality
Walker et al., 2017 [27]	Cross-functional collaboration metrics
Zeng et al., 2023 [28]	Leadership skills development, decision-making efficacy
Nowik et al., 2015 [29]	Success of mentorship programs
Karami, 2014 [30]	Accountability, accuracy in KPI tracking
Sreedharan et al., 2024 [31]	Data accessibility, transparency metrics

**Table 5:** Areas of intervention with the KPIs alongside the insight and the related study.

Key Area	Insight	References
Defining Clear Objectives and Selecting Relevant KPIs for the SRH Tool	Aligning the objectives of the SRH tool with the overarching goals of the healthcare organization ensures that the selected KPIs accurately measure performance, quality, and outcomes.	[22,23]
Establishing Transparent Communication Channels for the SRH Tool	Implementing various communication channels such as team meetings, digital platforms, and email updates facilitates the timely dissemination of information within healthcare organizations, specifically tailored to the implementation of the SRH tool.	[24,25]
Fostering a Collaborative Environment for the SRH Tool	Regular team meetings and cross-functional teams promote open dialogue, enabling diverse perspectives on KPI results and improvement strategies that are essential for successful healthcare delivery, including the integration and utilization of the SRH tool.	[26,27]
Implementing Leadership Development Programs for the SRH Tool	Leadership development programs equip leaders with the necessary skills to interpret KPI data and make informed decisions, complemented by mentorship programs for guidance and support to emerging leaders, thereby driving organizational success, including the implementation and utilization of the SRH tool.	[28,29]
Empowering Team Members for the SRH Tool	Delegating responsibilities related to KPI tracking and providing access to data and analysis tools fosters a sense of ownership and accountability among team members is crucial for organizational effectiveness, including the successful integration and utilization of the SRH tool.	[30,31]
Recognizing and Rewarding Achievements for the SRH Tool	Regular recognition and rewards based on KPI progress enhance morale and motivation within the team, contributing to a positive work culture and driving overall performance, including the successful implementation and utilization of the SRH tool.	[22,23]
Continuous Improvement and Adaptation for the SRH Tool	Establishing feedback loops and remaining agile allow organizations to adapt strategies and KPIs in response to changing organizational goals and performance data, which is essential for continuous improvement and organizational excellence, including the ongoing refinement and adaptation of the SRH tool.	[24,25]
Defining Clear Objectives and Selecting Relevant KPIs for the SRH Tool	Aligning the objectives of the SRH tool with the overarching goals of the healthcare organization ensures that the selected KPIs accurately measure performance, quality, and outcomes.	[22,23]

## Data Availability

Data is contained within the article.

## Supplementary Materials

The following supporting information can be downloaded at: https://www.mdpi.com/article/10.3390/jpm14070669/s1, Figure S1. Publications in the healthcare professionals sector focused on workload before and in the last five years. Figure S2. Publications in the healthcare professionals sector focused on competence before and in the last five years. Figure S3. Publications in the healthcare professionals sector focused on performance before and in the last five years. Figure S4. Representation of healthcare disciplines participating in the questionnaire. Box S1 composite keys used for the literature search.

## References

[1] Andersson B.T., Fridlund B., Elgán C., Axelsson A.B. (2008). Radiographers’ areas of professional competence related to good nursing care. Scand. J. Caring Sci..

[2] Taylor A., Bleiker J., Hodgson D. (2021). Compassionate communication: Keeping patients at the heart of practice in an advancing radiographic workforce. Radiography.

[3] Strudwick R.M., Day J. (2014). Interprofessional working in diagnostic radiography. Radiography.

[4] Hyde E., Hardy M. (2021). Patient centred care in diagnostic radiography (Part 1): Perceptions of service users and service deliverers. Radiography.

[5] Al-Shemmari A.F., Herbland A., Akudjedu T.N., Lawal O. (2022). Radiographer’s confidence in managing patients with claustrophobia during magnetic resonance imaging. Radiography.

[6] McNair H.A., Joyce E., O’Gara G., Jackson M., Peet B., Huddart R.A., Wiseman T. (2021). Radiographer-led online image guided adaptive radiotherapy: A qualitative investigation of the therapeutic radiographer role. Radiography.

[7] Brealey S., Scally A., Hahn S., Thomas N., Godfrey C., Coomarasamy A. (2005). Accuracy of radiographer plain radiograph reporting in clinical practice: A meta-analysis. Clin. Radiol..

[8] Mada M.O., Hindmarch P., Stirling J., Davies J., Brian D., Barnes A., Hammers A., Gulliver N., Herholz K., O’Brien J. (2020). Competencies and training of radiographers and technologists for PET/MR imaging—A study from the UK MR-PET network. Eur. J. Hybrid Imaging.

[9] Hall L.H., Johnson J., Watt I., Tsipa A., O’Connor D.B. (2016). Healthcare Staff Wellbeing, Burnout, and Patient Safety: A Systematic Review. PLoS ONE.

[10] Lu L., Ko Y.M., Chen H.Y., Chueh J.W., Chen P.Y., Cooper C.L. (2022). Patient Safety and Staff Well-Being: Organizational Culture as a Resource. Int. J. Environ. Res. Public Health.

[11] Copanitsanou P., Fotos N., Brokalaki H. (2017). Effects of work environment on patient and nurse outcomes. Br. J. Nurs. Mark. Allen Publ..

[12] Valentine N.M., Nash J., Hughes D., Douglas K. (2008). Achieving effective staffing through a shared decision-making approach to open-shift management. J. Nurs. Adm..

[13] Inoue M., Takano M., Ueno C., Mori M., Morimatsu Y., Matsumoto Y., Kushino N., Ishitake T. (2020). Advantages of the Variable Shift System, and Effective Use of Break Time to Better Support the Work Engagement of Nurses on Extended Day Shifts. Kurume Med. J..

[14] Batt A.M., Tavares W., Williams B. (2020). The development of competency frameworks in healthcare professions: A scoping review. Adv. Health Sci. Educ. Theory Pract..

[15] Koruca H.İ., Emek M.S., Gulmez E. (2023). Development of a new personalized staff-scheduling method with a work-life balance perspective: Case of a hospital. Ann. Oper. Res..

[16] Loef B., van der Beek A.J., Holtermann A., Hulsegge G., van Baarle D., Proper K.I. (2018). Objectively measured physical activity of hospital shift workers. Scand. J. Work. Environ. Health.

[17] Yezengaw T.Y., Debella A., Animen S., Aklilu A., Feyisa W., Hailu M., Sime B., Mohammed A., Deressa A., Mussa I. (2023). Clinical practice competence and associated factors among undergraduate midwifery and nursing sciences students at Bahir Dar city, Northwest Ethiopia. Ann. Med. Surg..

[18] Budoff M.J., Cohen M.C., Garcia M.J., Hodgson J.M., Hundley W.G., Lima J.A., Manning W.J., Pohost G.M., Raggi P.M., Rodgers G.P. (2005). ACCF/AHA clinical competence statement on cardiac imaging with computed tomography and magnetic resonance. Circulation.

[19] https://pubmed.ncbi.nlm.nih.gov/?term=%28healthcare+professional%5BTitle%2FAbstract%5D%29+AND+%28workload%5BTitle%2FAbstract%5D%29&sort=date&size=200.

[20] https://pubmed.ncbi.nlm.nih.gov/?term=%28healthcare+professional%5BTitle%2FAbstract%5D%29+AND+%28competence%5BTitle%2FAbstract%5D%29&sort=date&size=200.

[21] https://pubmed.ncbi.nlm.nih.gov/?term=%28healthcare+professional%5BTitle%2FAbstract%5D%29+AND+%28performance%5BTitle%2FAbstract%5D%29&sort=date&size=200.

[22] Abujudeh H.H., Kaewlai R., Asfaw B.A., Thrall J.H. (2010). Quality initiatives: Key performance indicators for measuring and improving radiology department performance. Radiographics.

[23] Nelson J., Ding A., Mann S., Parsons M., Samei E. (2022). Key Performance Indicators for Quality Imaging Practice: Why, What, and How. J. Am. Coll. Radiol..

[24] Di Costanzo D.J., Kumaraswamy L.K., Shuman J., Pavord D.C., Hu Y., Jordan D.W., Waite-Jones C., Hsu A. (2022). An introduction to key performance indicators for medical physicists. J. Appl. Clin. Med. Phys..

[25] European Society of Radiology (ESR) (2020). Performance indicators for radiation protection management: Suggestions from the European Society of Radiology. Insights Imaging.

[26] Schultz C.C., Shaffer S., Fink-Bennett D., Winokur K. (2016). Key Performance Indicators in the Evaluation of the Quality of Radiation Safety Programs. Health Phys..

[27] Walker E.A., Petscavage-Thomas J.M., Fotos J.S., Bruno M.A. (2017). Quality metrics currently used in academic radiology departments: Results of the QUALMET survey. Br. J. Radiol..

[28] Zeng A., Gu Y., Ma L., Tao X., Gao L., Li J., Wang H., Jiang Y. (2023). Development of Quality Indicators for the Ultrasound Department through a Modified Delphi Method. Diagnostics.

[29] Nowik P., Bujila R., Poludniowski G., Fransson A. (2015). Quality control of CT systems by automated monitoring of key performance indicators: A two-year study. J. Appl. Clin. Med. Phys..

[30] Karami M. (2014). A design protocol to develop radiology dashboards. Acta Inform. Med..

[31] Sreedharan J., Subbarayalu A.V., Kamalasanan A., Albalawi I., Krishna G.G., Alahmari A.D., Alsalamah J.A., Alkhathami M.G., Alenezi M., Alqahtani A.S. (2024). Key Performance Indicators: A Framework for Allied Healthcare Educational Institutions. Clinicoecon Outcomes Res..

[32] Portoghese I., Galletta M., Coppola R.C., Finco G., Campagna M. (2014). Burnout and workload among health care workers: The moderating role of job control. Saf. Health Work..

[33] Kovacs R., Lagarde M. (2022). Does high workload reduce the quality of healthcare? Evidence from rural Senegal. J. Health Econ..

[34] Rostami F., Babaei-Pouya A., Teimori-Boghsani G., Jahangirimehr A., Mehri Z., Feiz-Arefi M. (2021). Mental Workload and Job Satisfaction in Healthcare Workers: The Moderating Role of Job Control. Front. Public Health.

[35] Jose A., Tortorella G.L., Vassolo R., Kumar M., Mac Cawley A.F. (2022). Professional Competence and Its Effect on the Implementation of Healthcare 4.0 Technologies: Scoping Review and Future Research Directions. Int. J. Environ. Res. Public Health.

[36] Yaqoob Mohammed Al Jabri F., Kvist T., Azimirad M., Turunen H. (2021). A systematic review of healthcare professionals’ core competency instruments. Nurs. Health Sci..

[37] Jarva E., Oikarinen A., Andersson J., Pramila-Savukoski S., Hammarén M., Mikkonen K. (2024). Healthcare professionals’ digital health competence profiles and associated factors: A cross-sectional study. J. Adv. Nurs..

[38] Krijgsheld M., Tummers L.G., Scheepers F.E. (2022). Job performance in healthcare: A systematic review. BMC Health Serv. Res..

[39] Rana W., Mukhtar S., Mukhtar S. (2022). Job satisfaction, performance appraisal, reinforcement and job tasks in medical healthcare professionals during the COVID-19 pandemic outbreak. Int. J. Health Plan. Manag..

[40] Crawshaw J., Meyer C., Antonopoulou V., Antony J., Grimshaw J.M., Ivers N., Konnyu K., Lacroix M., Presseau J., Simeoni M. (2023). Identifying behaviour change techniques in 287 randomized controlled trials of audit and feedback interventions targeting practice change among healthcare professionals. Implement. Sci..

[41] Chandramohan A., Krothapalli V., Augustin A., Kandagaddala M., Thomas H.M., Sudarsanam T.D., Jagirdar A., Govil S., Kalyanpur A. (2023). Teleradiology and technology innovations in radiology: Status in India and its role in increasing access to primary health care. Lancet Reg. Health Southeast. Asia.

[42] Lepri G., Oddi F., Gulino R.A., Giansanti D. (2024). Reimagining Radiology: A Comprehensive Overview of Reviews at the Intersection of Mobile and Domiciliary Radiology over the Last Five Years. Bioengineering.

[43] Giansanti D., Rossi I., Monoscalco L. (2021). Lessons from the COVID-19 Pandemic on the Use of Artificial Intelligence in Digital Radiology: The Submission of a Survey to Investigate the Opinion of Insiders. Healthcare.

