# Supplementary material for: Precision Workforce Management for Radiographers: Monitoring and Managing Competences with an Automatic Tool
Source: J Pers Med. 2024 Jun 21;14(7):669. doi: 10.3390/jpm14070669 (PMC11278459; doi:10.3390/jpm14070669)
Supplement: Supplementary file 1 [file jpm-14-00669-s001.zip › jpm-3017576-supplementary.pdf]

SUPPLEMENTARY MATERIAL

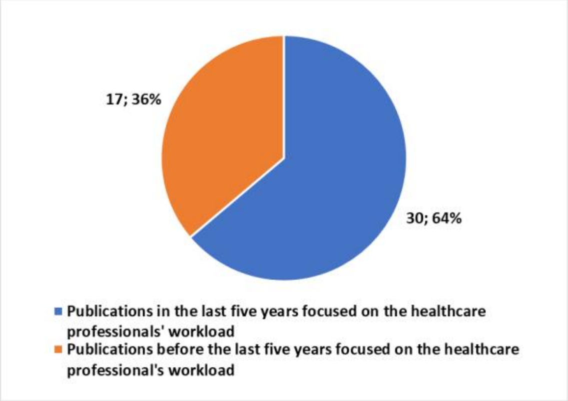

Figure s. 1. Publications in the healthcare professionals sector focused on workload before and in the last five years.

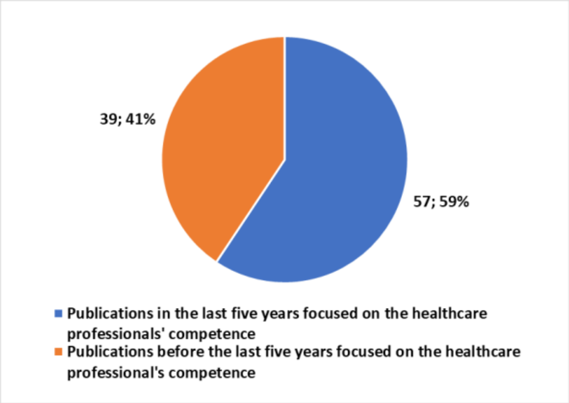

Figure s. 2. Publications in the healthcare professionals sector focused on competence before and in the last five years.

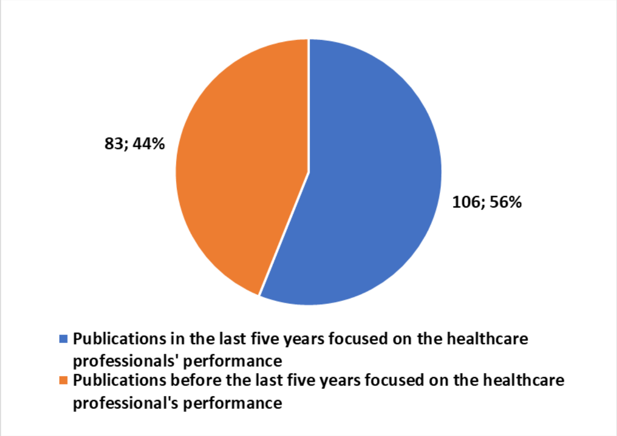

Figures. 3. Publications in the healthcare professionals sector focused on performance before and in the last five years.

.

Box s. 1 composite keys used for the literature search

(healthcare professional[Title/Abstract]) AND (workload[Title/Abstract])
(healthcare professional[Title/Abstract]) AND (competence[Title/Abstract])
(healthcare professional[Title/Abstract]) AND (performance[Title/Abstract])

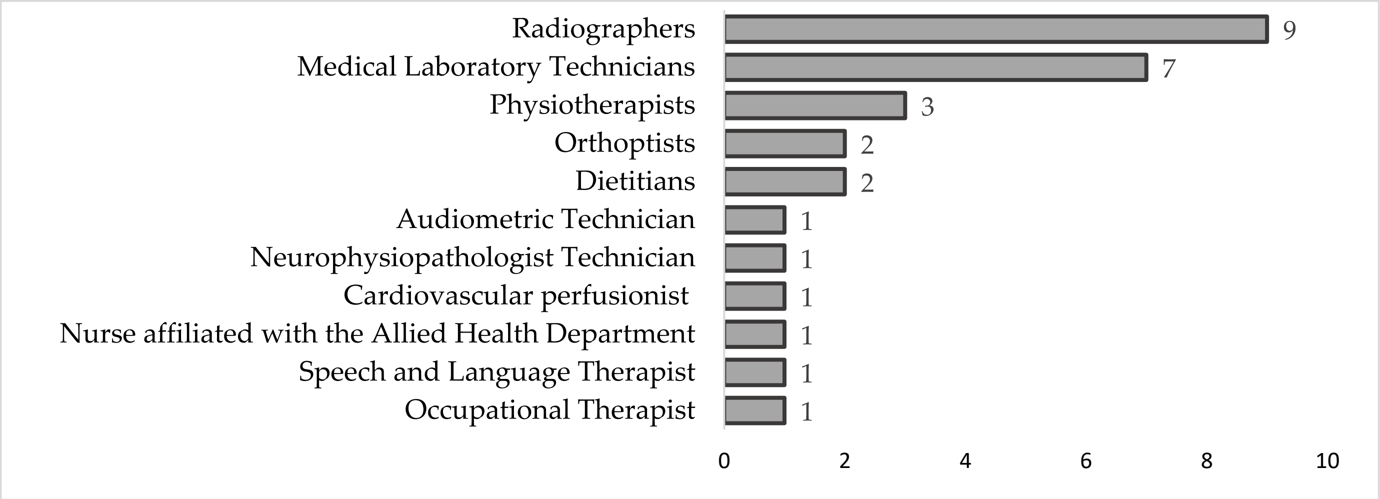

Figure s.4 Representation of healthcare disciplines participating in the questionnaire
